# Aberrations in circulating inflammatory cytokine levels in patients with Down syndrome: a meta-analysis

**DOI:** 10.18632/oncotarget.21060

**Published:** 2017-09-19

**Authors:** Yan Zhang, Meng Che, Jing Yuan, Yun Yu, Chang Cao, Xiao-Yan Qin, Yong Cheng

**Affiliations:** ^1^ Center on Translational Neuroscience, College of Life and Environmental Sciences, Minzu University of China, Beijing 100081, China

**Keywords:** cytokine, inflammation, Down syndrome, meta-analysis, systematic review

## Abstract

Evidence suggests that immune system alterations in Down syndrome (DS) may be early events that drive neuropathological and cognitive changes of Alzheimer's disease. The primary objective of this meta-analysis was to investigate whether there is an abnormal cytokine profile in DS patients when compared with healthy control (HC) subjects. A systematic search of Pubmed and Web of Science identified 19 studies with 957 DS patients and 541 HC subjects for this meta-analysis. Random effects meta-analysis demonstrated that patients with DS had significantly increased circulating tumor necrosis factor-α (Hedges’ g = 1.045, 95% confidence interval (CI) = 0.192 to 1.898, *p* = 0.016), interleukin (IL)-1β (Hedges’ g = 0.696, 95% confidence CI = 0.149 to 1.242, *p* = 0.013), interferon-γ (Hedges’ g = 0.978, 95% CI = 0.417 to 1.539, *p* = 0.001) and neopterin (Hedges’ g = 0.815, 95% CI = 0.423 to 1.207, *p* < 0.001) levels compared to HC subjects. No significant differences were found between patients with DS and controls for concentrations of IL-4, IL-6, IL8 and IL-10. In addition, most of the cytokine data in this meta-analysis were from children with DS and HC, and subgroup analysis showed that children with DS had elevated tumor necrosis factor-α, IL-1β and interferon-γ levels when compared with controls. Taken together, these results demonstrated that patients (children) with DS are accompanied by increased circulating cytokine tumor necrosis factor-α, IL-1β and interferon-γ levels, strengthening the clinical evidence that patients (children) with DS are accompanied by an abnormal inflammatory response.

## INTRODUCTION

Down syndrome (DS), also known as trisomy 21, is one of the most common chromosomal abnormalities in humans, which is the prevailing cause of mental retardation [1]. It is estimated that more than 5 million people worldwide are affected by DS, and the prevalence of the disease varies between 250,000 and 400,000 in the United States [2]. In addition to the mental disabilities caused early in life, virtually all patients with DS develop Alzheimer’s-disease (AD) neuropathological changes in the brain by their 40 s, and at least 70% will develop dementia by age 55–60 s [1, 3–5]. Unfortunately, there are no treatments to delay or prevent the progression of dementia in adult DS patients. Given the ability to identify DS patients at or before birth and the high incidence of AD among adults with DS, improved understanding of early events that contribute to and/or promote the neuropathophysiological progression of AD in DS may provide early intervention or prevention of AD in this population, and may also shed light on the ways in which neurodegeneration occurs in sporadic AD.

One early event that may contribute to the progression of dementia in DS patients is amyloid beta deposition in the brains, as amyloid beta plagues have been noticed in some DS children [1]. Another possible pathogenic link between DS and AD is the dysregulation of immune response in those patients both in central and peripheral, this is evidenced by glial cell activation with excessive expression of inflammatory markers such as chromosome 2 gene product IL-1 and chromosome 21 gene product S100B in DS and AD brains [6, 7]. The pro-inflammatory cytokines TNF-α, IL-1β, IL-6 and IFN-γ are considered to play key roles in inflammatory responses. It has been reported that macrophages and T cells secret IL-6 to stimulate the inflammatory responses, while IL-1β promotes B cell maturation and induces immunoglobulin production, which eventually leads to inflammation [8]. Therefore, a substantial number of studies have analyzed levels of these inflammatory cytokines in patients with AD and DS, in hope of better understanding of the etiology of the diseases and potentially use cytokines as biomarkers for disease progression. Although clinical data were not always consistent across studies, results from a meta-analysis demonstrated that peripheral blood inflammatory cytokines including tumor necrosis factor (TNF) -α, interleukin (IL)-1β and IL-6 were significantly increased in patients with AD compared with healthy control (HC) subjects [9]. However, the changes of inflammatory cytokines in DS patients were unclear due to the inconsistent data for individual cytokine and between studies [10–14]. Therefore, a meta-analysis on this subject is necessary to address the inconsistency in clinical data, especially the inflammatory cytokine changes in children with DS.

## MATERIALS AND METHODS

We performed meta-analysis in this study adhered to the guidelines that are recommended by the PRISMA statement (Preferred Reporting Items for Systematic reviews and Meta-Analysis) [15] .

Two independent investigators performed a systematic review of peer reviewed English articles from databases of Pubmed and Web of Science through May, 2017. The database search term was: (inflammation or cytokine or chemokine or tumor necrosis factor or interleukin or interferon or neopterin or C-reactive protein) and (down syndrome or trisomy 21), no year limitation was applied. Original clinical studies that reported data on circulating cytokine concentrations in down syndrome patients and controls were included. Excluded criteria were: (1) *in vitro* studies with reported stimulated or unstimulated levels of cytokines; (2) samples were same cohort with other studies; (3) Cytokines were not analyzed in at least three studies.

### Data extraction

The data were extracted by two independent investigators. Data on sample size, mean cytokine concentration, standard deviation (SD) and *p* value were extracted as primary outcomes. Data for potential moderator analysis of age, gender, sampling source and assay type were also extracted (see Table 1). It should be noted that the “children” in this study were individuals aged between 0–18 years as PubMed classified.

**Table 1:** Characteristics of included studies measuring peripheral circulating cytokine concentration

Study/Year	Cytokines Measured	Country	Samples (DS/Control)	Gender (% Male) (DS/Control)	Mean Age (DS/Control)	Sample Source	Assay type
Barr-Agholme et al. 1997	IL-1β	Sweden	15/15	NA	12.1/13.8	GCF	ELISA
Broers et al. 2012	IFN-γ, IL-1β, IL-6, IL-8, IL-10, TNF-α	Netherlands	61/57	64/40	7.8/9.3	Blood	Cytometric Bead Assay, ELISA
Carta et al. 2002	IFN-γ, IL-6, TNF-α	Italy	19/19	68.4/68.4	30.11/30.27	Blood	ELISA
Cattell et al. 1989	Neopterin	UK	53/32	NA	NA	Urine	HPLC
Cetiner et al. 2010	IL-1β, IL-4, IL-6, IL-8, IL-10, TNF-α	Turkey	32/32	43.8/56.2	3.9/4.5	Blood	ELISA
Coppus et al. 2010	Neopterin	Netherlands	401/48	62.3/66.7	52/50.2	Blood	HPLC
Dogliotti et al. 2010	IL-6	Italy	50/30	NA	NA	Blood	biochip array analyzer
Licastro et al. 2005	IL-6, Neopterin	Italy	40/20	NA	NA	Blood	ELISA
Mehta et al. 2007	Neopterin	USA	35/34	80.0/58.8	7.17/10.71	Blood	ELISA
Mehta et al. 2005	Neopterin	USA	38/37	50/51.4	45/44	Blood	ELISA
Nelson et al. 2006	IL-8	USA	46/40	NA	NA	Blood	xMAP Luminex
Parisotto et al. 2015	IL-1β, TNF-α	Brazil	21/18	55.6/57.1	7.7/6.7	Blood	ELISA
Rodrigues et al. 2014	IFN-γ, IL-1β, IL-6, IL-10, TNF-α	Brazil	23/23	47.8/39.1	28.3/27.7	Blood	ELISA
Rostami et al. 2012	IFN-γ , IL-10, TNF-α	Iran	24/24	45.8/NA	5.75/5.75	Blood	ELISA
Shimada et al. 2007	IFN-γ, IL-1β, IL-4, IL-6, IL-8, IL-10, TNF-α	Japan	15/10	NA	NA	Blood	Multiplex Suspension Array System
Smigielska-Kuzia et al. 2010	IL-4, IL-10	Poland	5/10	NA	NA	Blood	ELISA
Torre et al. 1995	IFN-γ	Italy	12/20	NA	38.3/34.9	Blood	ELISA
Tsilingaridis et al. 2012	IFN-γ, IL-1β, IL-4, IL-6, IL-10, TNF-α	Sweden	24/29	54.2/48.3	16.4/16.4	GCF	Bio-Plex Cytokine Assay
Zaki et al. 2017	IL-6, TNF-α	Egypt	43/43	46.5/51.2	6.4/6.3	Blood	ELISA

Abbreviations: DS, Down syndrome; IL, interleukin; GCF, gingival crevicular fluid; ELISA, enzyme linked immunosorbent assay; IFN-γ, interferon-γ; TNF-α, tumor necrosis factor-α; HPLC, high performance liquid chromatography; NA, not available.

### Statistical analysis

We used Comprehensive Meta-Analysis Version 2 software (Biostat Inc., Englewood, NJ, USA) to perform all the statistical analyses. Sample size, mean concentration and standard deviation (SD) were primarily used to generate effective size, in some cases effective sizes were generated by sample size and *p* value when mean concentration and SD were not available. Effective size was calculated as standardized mean difference of cytokine concentrations between DS patients and controls, and converted to Hedges'g which provides an unbiased ES adjusted for sample size [16]. Random effects meta-analysis was chosen in this study because we hypothesized that both within-study variance and between-study variance affected the true effective size. In addition, we performed sensitivity analysis by removing one study at a time to assess whether a single study could influence the statistical significance of the meta-analysis.

We assessed between-study heterogeneity by Cochrane's *Q* test and I^2^ index as described previously [17]. Statistical difference for the Cochrane's *Q* test was set at *p* < 0.1; I^2^ of 0.75, 0.50 and 0.25 indicated high, moderate and small levels of heterogeneity, respectively. The potential moderating effects of age, gender and sample size on the meta-analysis were analyzed by unrestricted maximum-likelihood random-effects meta-regressions of effective size. Furthermore, Egger's test was used to test publication bias, which assesses the funnel plot asymmetry.

*P* < 0.05 was considered statistical significant in this study except where noted.

## RESULTS

**Figure 1:**
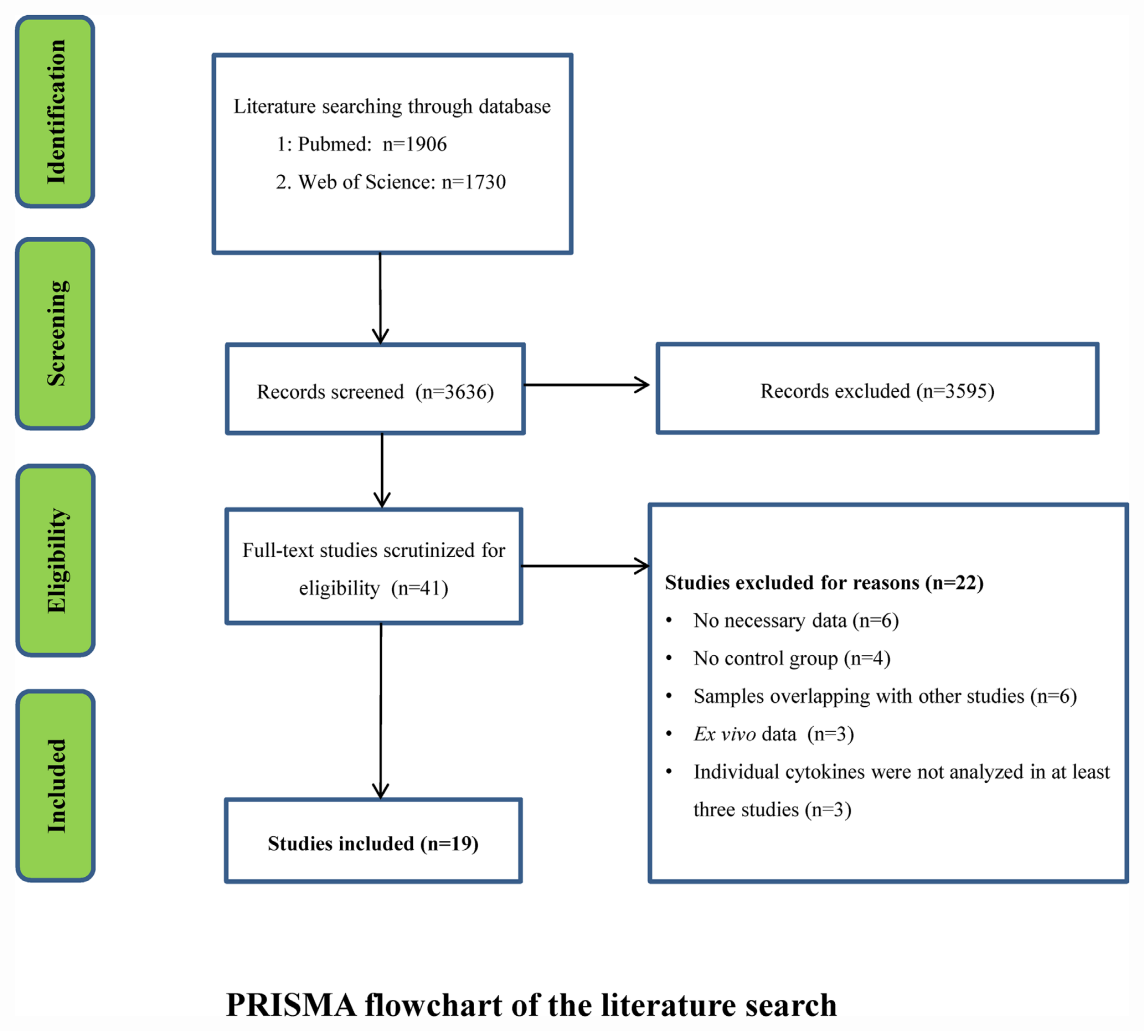
PRISMA flowchart of the literature search

Systematic review of the literature identified 1906 records from Pubmed and 1730 records from Web of Science. Scanning of titles and abstracts resulted in identification of 41 articles for full text scrutiny. Several studies were excluded because: *in vitro* blood cytokine levels were reported (3 studies); no necessary data (6 studies); lack of control group (4 studies); samples were overlapping with other studies (6 studies); Cytokines were not analyzed in at least three studies (3 studies). Therefore, a total of 19 studies encompassing 957 DS patients and 541 HC subjects were included in the meta-analysis [10–14, 18–31] (Flowchart see Figure 1).

### Main association of DS with cytokines

**Figure 2:**
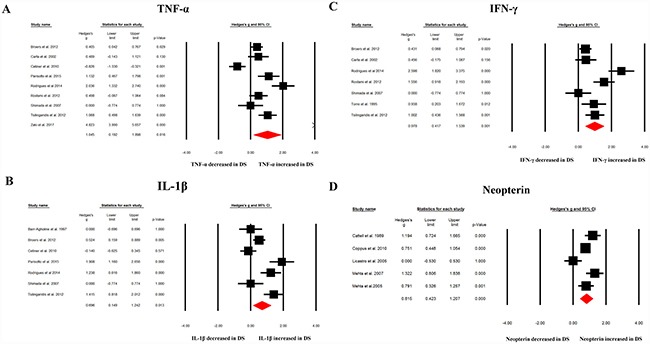
Forest plot displaying random effects meta-analysis results of the association between TNF-α (**A**), IL-1β (**B**), IFN-γ (**C**), neopterin (**D**) and DS. The sizes of the squares are proportional to study weights.

Random effects meta-analysis demonstrated that patients with DS had significantly higher circulating inflammatory marker levels compared with HC subjects for TNF-α (Hedges’ g = 1.045, 95% confidence interval (CI) = 0.192 to 1.898, *p* = 0.016), IL-1β (Hedges’ g = 0.696, 95% CI = 0.149 to 1.242, *p* = 0.013), IFN-γ (Hedges’ g = 0.978, 95% CI = 0.417 to 1.539, *p* = 0.001) and neopterin (Hedges’ g = 0.815, 95% CI = 0.423 to 1.207, *p* < 0.001), as shown in Table 2 and Figure 2. However, circulating IL-4, IL-6, IL8 or IL-10 levels did not differentiate between DS patients and HC subjects (Table 2).

**Table 2:** Summary of comparative outcomes for peripheral circulating cytokine measurements

Cytokine	No.of studies	No.with DS/Controls	Main effects			Heterogeneity				Publication Bias	
			Hedges g (95% CI)	*z* Score	*P* Value	Q Statistic	df	*P* Value	I^2^ Statistic	Egger Intercept	*P* Value
IL-1β	7	191/184	0.696 (0.149 to 1.242)	2.497	.013	37.211	6	< .001	83.876	2.41	.55
IL-4	4	76/81	1.041 (–0.394 to 2.477)	1.422	.155	44.097	3	< .001	93.197	–4.88	.71
IL-6	9	307/263	0.665 (–0.260 to 1.591)	1.409	.159	190.392	8	< .001	95.798	–1.42	.85
IL-8	4	154/139	0.291 (–0.249 to 0.831)	1.056	.291	14.706	3	.002	79.600	–4.24	.47
IL-10	6	179/175	–0.169 (–0.970 to 0.633)	–0.413	.68	63.282	5	< .001	92.099	–4.76	.44
INF-γ	7	178/182	0.978 (0.417 to 1.539)	3.417	.001	35.686	6	< .001	83.187	3.85	.29
Neopterin	5	567/171	0.815 (0.423 to 1.207)	4.075	< .001	15.471	4	.004	74.146	0.21	.97
TNF-α	9	262/255	1.045 (0.192 to 1.898)	2.4	.016	152.717	8	< .001	94.762	8.95	.13

Abbreviations: CI, confidence interval; DS, Down syndrome; TNF, tumor necrosis factor-α; IFN, interferon; IL, interleukin; df, degree of freedom; Q, Cochran's *Q* test; *z* (test of null hypothesis); *p* (statistical significance); I^2^ (heterogeneity level).

### Investigation of heterogeneity

Significant heterogeneity was found for all eight cytokines analyzed in this meta-analysis. TNF-α, IL-1β, IL-4, IL-6, IL8, IL-10 and IFN-γ showed high levels of heterogeneity, and neopterin showed moderate levels of heterogeneity (Table 2).

### Subgroup analysis

We then performed subgroup analysis to test whether theoretically relevant categorical variables moderate the between-study heterogeneity. Since most of the studies measured cytokine levels in children with DS, we therefore performed meta-analysis to analyze cytokine changes in children with DS. As shown in Supplementary Figure 1, children with DS had elevated TNF-α (7 studies, Hedges’ g = 0.988, 95% CI = –0.043 to 2.02, *p* = 0.06), IL-1β (6 studies, Hedges’ g = 0.606, 95% CI = 0.003 to 1.209, *p* = 0.049) and IFN-γ (4 studies, Hedges’ g = 0.754, 95% CI = 0.162 to 1.346, *p* = 0.013) levels when compared with control children, although it did not reach statistical significance for TNF-α. However, between-study heterogeneity remains high for TNF-α (Q_6_ = 13.202; *p* < 0.001; I^2^ = 95.646), IL-1β (Q_5_ = 32.839; *p* < 0.001; I^2^ = 84.774) and IFN-γ (Q_3_ = 13.26; *p* = 0.004; I^2^ = 77.376).

We also analyzed blood cytokine levels in DS patients, considering most of the samples in the included studies in this meta-analysis were from blood. The meta-analysis suggested that blood TNF-α (8 studies, Hedges’ g = 1.045, 95% CI = 0.077 to 2.013, *p* = 0.034), IL-1β (5 studies, Hedges’ g = 0.683, 95% CI = 0.027 to 1.34, *p* = 0.041), IFN-γ (6 studies, Hedges’ g = 0.978, 95% CI = 0.306 to 1.651, *p* = 0.004) and neopterin (4 studies, Hedges’ g = 0.722, 95% CI = 0.271 to 1.173, *p* = 0.002) levels were significantly increased in DS patients compare with controls. Still, we found high levels of heterogeneity for TNF-α (Q_7_ = 150.973; *p* < 0.001; I^2^ = 95.363), IL-1β (Q_4_ = 27.102; *p* < 0.001; I^2^ = 85.241) and IFN-γ (Q_5_ = 35.291; *p* < 0.001; I^2^ = 85.832).

### Meta-regression analyses

We next performed meta-regression analyses on the association between TNF-α and DS, and revealed that age and gender of patients, and sample size (*p* > 0.05 in all the analyses) had no moderating effects on the outcome of the meta-analysis.

### Sensitivity analyses

Sensitivity analyses suggested that no single study significantly influenced the significant difference on circulating TNF-α, IFN-γ, IL-1β and neopterin levels between DS patients and HC subjects.

### Publication bias

Furthermore, no publication bias was found for the studies included in the meta-analysis, as suggested by funnel plots (Supplementary Figure 2) and Egger's test (*p* > 0.1 in all the analyses, see Table 2).

## DISCUSSION

To the best of our knowledge, this is the first meta-analysis undertaken to investigate alterations of circulating inflammatory cytokines in patients with DS compared with controls. It reports significant elevations of peripheral circulating pro-inflammatory cytokines TNF- α, IL-1β and IFN-γ in DS patients compared with HC subjects. Levels of another inflammatory marker neopterin, which is synthesized by human macrophages upon stimulation with the cytokine IFN-γ, were also elevated in patients with DS. The effective size associated with the results of TNF- α, IFN-γ and neopterin were large, and the effective size for IL-1β was medium. In addition, sensitivity analysis demonstrated that the significant associations between TNF- α, IFN-γ, IL-1β and neopterin levels and DS were not influenced by any single study, suggesting the robustness of the outcome of the meta-analysis. However, sensitivity analysis suggested that one study [11] significantly influenced the outcome of the meta-analysis for IL-6. Removing this outlier, IL-6 levels were significantly associated with DS, with large effective size (8 studies, Hedges’ g = 1.114, 95% CI = 0.46 to 1.767, *p* = 0.001). Thus, more work would be necessary to study the association between IL-6 and DS. Although clinical data from literature showed inconsistency in individual cytokines and between studies, results from our meta-analysis provided strong clinical evidence of a heightened pro-inflammatory cytokine profile in patients with DS.

Accumulating evidence suggest that reactive microglial cells in central nervous system and aberrant levels of inflammatory cytokines may contribute to the onset and progression of major neurological disorders, these include AD [32, 33] and Parkinson’ disease (PD) [34, 35]. Previous meta-analyses have been performed for peripheral blood cytokine levels in AD [9] and PD [36]. Similar to the findings of our present meta-analysis in DS, levels of blood TNF-α and IL-1β were elevated in patients with AD and PD, suggesting that the elevations of circulating inflammatory cytokine TNF-α and IL-1β are not specific in DS. However, the inflammatory cytokine IFN-γ, which the present meta-analysis showed highly significant association with DS, was not reported to be associated with AD and PD in the respective meta-analyses. Consistently, this meta-analysis found that the IFN-γ stimulated inflammatory marker neopterin levels were increased in DS patients. The strong evidence of hyperactivity of IFN-γ pathway found in DS patients are in consistent with the fact that genes encoding IFN families and receptors are located on chromosome 21 [37, 38]. Since it is known that activation of IFN receptors induce pro-inflammatory cytokines, including expression of TNF-α and IL-1β [1], it is natural to assume that IFN signaling plays a critical role in regulating immune response in DS, and may serve as accelerators of AD neuropathogenesis in DS.

For those cytokines significantly associated with DS, we found high levels of between-study heterogeneity for TNF-α, IL-1β and IFN-γ. Although this meta-analysis used sub-group and meta-regression analyses to adjust for potential confounders, none of the theoretically relevant continuous and categorical variables that we analyzed could explain the heterogeneity. The sub-group analysis showed that IL-1β and IFN-γ levels were significantly increased in children with DS when compared with controls, but the high levels of heterogeneity among studies were not reduced in this sub-group. Similarly, the between study heterogeneity for the blood inflammatory marker TNF-α, IL-1β, IFN-γ and neopterin were not reduced. Although it is unclear what caused the between-study heterogeneity for cytokines analyzed in this meta-analysis, clinical and methodological moderators that were not assessed in our meta-analysis may have contributed to the observed high levels of between-study heterogeneity. These include various methodological issues such as handling of samples (the timing of sampling, the time between sample collection and processing/analysis, storage conditions, and the freeze–thaw cycle effect). Furthermore, operator reliability in different laboratories and variability in assay procedure using kits from different manufacturers may also confound the results. In terms of clinical moderators, several confounders such as unreported medication, disease comorbidity and sleep problems could be potential sources to explain the between-study heterogeneity. Although most of the studies included in this meta-analysis did not include or report DS patients with disease comorbidity, Tsilingaridis et al. reported that some children with DS had congenital cardiac malformations, epilepsy or autism [30], but it is unclear whether the disease comorbidity affected the inflammatory response in the patients. Taken together, these highlight the need for continued work on aberrant regulations of cytokines in DS patients to better understand the altered immune response in those patients.

Although this meta-analysis provides strong clinical evidence of a heightened pro-inflammatory response in DS, with increased circulating TNF-α, IL-1β, IFN-γ and neopterin levels, this study has several limitations. First, the meta-analysis of circulating cytokine levels in patients with DS compared with HC subjects provides us pooled results originating from cross-sectional studies. Therefore, it is unclear whether the inflammatory cytokines contribute to the development of AD in DS patients, longitudinal studies maybe necessary to reveal the potential of the cytokines to serve as biomarkers for disease progression of AD in DS, and subsequently develop targeted intervention to prevent or delay the onset of AD. Second, this meta-analysis did not find altered levels of pro-inflammatory cytokines IL-6 and IL-8, and anti-inflammatory IL-4 and IL-10 levels in patients with DS compared with HC subjects, the limitation of this meta-analysis is that the limited number of studies with a smaller sample size may have made observation of significant associations difficult for cytokine IL-4, IL-8, and IL-10. In addition, other inflammatory markers, including chemokines and C-reactive protein were not included in this meta-analysis, this is because these inflammatory marker data were not reported in at least three studies. However, Dogliotti et al. showed that blood chemokine (C-C motif) ligand-2 (CCL-2) levels were significantly elevated in patients with DS [22]. Moreover, data from Carta et al. indicated that patients with DS had increased blood CCL-3 levels when compared with controls, but CCL-4 and CCL-5 levels did not differentiate between cases and controls [19]. For C-reactive protein, one study reported normal blood levels of C-reactive protein in patients with DS [39], whereas another study demonstrated that the levels of this inflammatory marker were elevated in the blood of DS patients [23]. Therefore, patients with DS may have abnormal chemokine and C-reactive protein profile in circulation, but future studies are necessary to substantiate this idea.

In conclusion, this meta-analysis demonstrated elevated peripheral circulating TNF-α, IL-1β, IFN-γ and neopterin in patients with DS. The finding strengthen the clinical evidence that patients (children) with DS is accompanied by an aberrant inflammatory response.

## SUPPLEMENTARY MATERIALS FIGURES



## References

[1] Wilcock DM, Griffin WS (2013). Down’s syndrome, neuroinflammation, and Alzheimer neuropathogenesis. J Neuroinflammation.

[2] Hartley D, Blumenthal T, Carrillo M, DiPaolo G, Esralew L, Gardiner K, Granholm AC, Iqbal K, Krams M, Lemere C, Lott I, Mobley W, Ness S (2015). Down syndrome and Alzheimer’s disease: Common pathways, common goals. Alzheimers Dement.

[3] Lai F, Williams RS (1989). A prospective study of Alzheimer disease in Down syndrome. Arch Neurol.

[4] Mann DM (1988). The pathological association between Down syndrome and Alzheimer disease. Mech Ageing Dev.

[5] Wisniewski KE, Wisniewski HM, Wen GY (1985). Occurrence of neuropathological changes and dementia of Alzheimer’s disease in Down’s syndrome. Ann Neurol.

[6] Griffin WS, Stanley LC, Ling C, White L, MacLeod V, Perrot LJ, White CL, Araoz C (1989). Brain interleukin 1 and S-100 immunoreactivity are elevated in Down syndrome and Alzheimer disease. Proc Natl Acad Sci USA.

[7] Royston MC, McKenzie JE, Gentleman SM, Sheng JG, Mann DM, Griffin WS, Mrak RE (1999). Overexpression of s100beta in Down’s syndrome: correlation with patient age and with beta-amyloid deposition. Neuropathol Appl Neurobiol.

[8] Dinarello CA (2000). Proinflammatory cytokines. Chest.

[9] Swardfager W, Lanctot K, Rothenburg L, Wong A, Cappell J, Herrmann N (2010). A meta-analysis of cytokines in Alzheimer’s disease. Biol Psychiatry.

[10] Broers CJ, Gemke RJ, Weijerman ME, van der Sluijs KF, van Furth AM (2012). Increased pro-inflammatory cytokine production in Down Syndrome children upon stimulation with live influenza A virus. J Clin Immunol.

[11] Cetiner S, Demirhan O, Inal TC, Tastemir D, Sertdemir Y (2010). Analysis of peripheral blood T-cell subsets, natural killer cells and serum levels of cytokines in children with Down syndrome. Int J Immunogenet.

[12] Nateghi Rostami M, Douraghi M, Miramin Mohammadi A, Nikmanesh B (2012). Altered serum pro-inflammatory cytokines in children with Down’s syndrome. Eur Cytokine Netw.

[13] Rodrigues R, Debom G, Soares F, Machado C, Pureza J, Peres W, de Lima Garcias G, Duarte MF, Schetinger MR, Stefanello F, Braganhol E, Spanevello R (2014). Alterations of ectonucleotidases and acetylcholinesterase activities in lymphocytes of Down syndrome subjects: relation with inflammatory parameters. Clin Chim Acta.

[14] Shimada A, Hayashi Y, Ogasawara M, Park MJ, Katoh M, Minakami H, Kitoh T, Kojima S, Kawa K, Kimura H (2007). Pro-inflammatory cytokinemia is frequently found in Down syndrome patients with hematological disorders. Leuk Res.

[15] Liberati A, Altman DG, Tetzlaff J, Mulrow C, Gotzsche PC, Ioannidis JP, Clarke M, Devereaux PJ, Kleijnen J, Moher D (2009). The PRISMA statement for reporting systematic reviews and meta-analyses of studies that evaluate health care interventions: explanation and elaboration. J Clin Epidemiol.

[16] Qin XY, Cao C, Cawley NX, Liu TT, Yuan J, Loh YP, Cheng Y (2017). Decreased peripheral brain-derived neurotrophic factor levels in Alzheimer’s disease: a meta-analysis study (N = 7277). Mol Psychiatry.

[17] Qin XY, Feng JC, Cao C, Wu HT, Loh YP, Cheng Y (2016). Association of Peripheral Blood Levels of Brain-Derived Neurotrophic Factor With Autism Spectrum Disorder in Children: A Systematic Review and Meta-analysis. JAMA Pediatr.

[18] Barr-Agholme M, Krekmanova L, Yucel-Lindberg T, Shinoda K, Modeer T (1997). Prostaglandin E2 level in gingival crevicular fluid from patients with Down syndrome. Acta Odontol Scand.

[19] Carta MG, Serra P, Ghiani A, Manca E, Hardoy MC, Del Giacco GS, Diaz G, Carpiniello B, Manconi PE (2002). Chemokines and pro-inflammatory cytokines in Down’s syndrome: an early marker for Alzheimer-type dementia?. Psychother Psychosom.

[20] Cattell RJ, Hamon CG, Corbett JA, Lejeune J, Blair JA (1989). Neopterin: biopterin ratios in Down’s syndrome. J Neurol Neurosurg Psychiatry.

[21] Coppus AM, Fekkes D, Verhoeven WM, Tuinier S, van Duijn CM (2010). Plasma levels of nitric oxide related amino acids in demented subjects with Down syndrome are related to neopterin concentrations. Amino Acids.

[22] Dogliotti G, Galliera E, Licastro F, Corsi MM (2010). Age-related changes in plasma levels of BDNF in Down syndrome patients. Immun Ageing.

[23] Licastro F, Chiappelli M, Ruscica M, Carnelli V, Corsi MM (2005). Altered cytokine and acute phase response protein levels in the blood of children with Downs syndrome: relationship with dementia of Alzheimer’s type. Int J Immunopathol Pharmacol.

[24] Mehta PD, Capone G, Jewell A, Freedland RL (2007). Increased amyloid beta protein levels in children and adolescents with Down syndrome. J Neurol Sci.

[25] Mehta PD, Patrick BA, Dalton AJ, Patel B, Mehta SP, Pirttila T, Coyle PK (2005). Increased serum neopterin levels in adults with Down syndrome. J Neuroimmunol.

[26] Nelson PG, Kuddo T, Song EY, Dambrosia JM, Kohler S, Satyanarayana G, Vandunk C, Grether JK, Nelson KB (2006). Selected neurotrophins, neuropeptides, and cytokines: developmental trajectory and concentrations in neonatal blood of children with autism or Down syndrome. Int J Dev Neurosci.

[27] Parisotto EB, Giaretta AG, Zamoner A, Moreira EA, Frode TS, Pedrosa RC, Filho DW (2015). Persistence of the benefit of an antioxidant therapy in children and teenagers with Down syndrome. Res Dev Disabil.

[28] Smigielska-Kuzia J, Bockowski L, Sobaniec W, Sendrowski K, Zelazowska-Rutkowska B, Cholewa M (2010). Anti-inflammatory plasma cytokines in children and adolescents with Down syndrome. Folia Histochem Cytobiol.

[29] Torre D, Broggini M, Zeroli C, Agrifoglio L, Botta V, Casalone R, Ferrario G (1995). Serum levels of gamma interferon in patients with Down’s syndrome. Infection.

[30] Tsilingaridis G, Yucel-Lindberg T, Modeer T (2012). T-helper-related cytokines in gingival crevicular fluid from adolescents with Down syndrome. Clin Oral Investig.

[31] Zaki ME, El-Bassyouni HT, Tosson AM, Youness E, Hussein J (2017). Coenzyme Q10 and pro-inflammatory markers in children with Down syndrome: clinical and biochemical aspects. J Pediatr (Rio J).

[32] Brosseron F, Krauthausen M, Kummer M, Heneka MT (2014). Body fluid cytokine levels in mild cognitive impairment and Alzheimer’s disease: a comparative overview. Mol Neurobiol.

[33] Heneka MT, Carson MJ, El Khoury J, Landreth GE, Brosseron F, Feinstein DL, Jacobs AH, Wyss-Coray T, Vitorica J, Ransohoff RM, Herrup K, Frautschy SA, Finsen B (2015). Neuroinflammation in Alzheimer’s disease. Lancet Neurol.

[34] More SV, Kumar H, Kim IS, Song SY, Choi DK (2013). Cellular and molecular mediators of neuroinflammation in the pathogenesis of Parkinson’s disease. Mediators Inflamm.

[35] Vivekanantham S, Shah S, Dewji R, Dewji A, Khatri C, Ologunde R (2015). Neuroinflammation in Parkinson’s disease: role in neurodegeneration and tissue repair. Int J Neurosci.

[36] Qin XY, Zhang SP, Cao C, Loh YP, Cheng Y (2016). Aberrations in Peripheral Inflammatory Cytokine Levels in Parkinson Disease: A Systematic Review and Meta-analysis. JAMA Neurol.

[37] Boselli D, Ragimbeau J, Orlando L, Cappello P, Capello M, Ambrogio C, Chiarle R, Marsili G, Battistini A, Giovarelli M, Pellegrini S, Novelli F (2010). Expression of IFNgammaR2 mutated in a dileucine internalization motif reinstates IFNgamma signaling and apoptosis in human T lymphocytes. Immunol Lett.

[38] Kim SH, Cohen B, Novick D, Rubinstein M (1997). Mammalian type I interferon receptors consists of two subunits: IFNaR1 and IFNaR2. Gene.

[39] He J, Li T, Chen J, Liu Y, Xiong F, Yang J, Song C (2016). Plasma antioxidant enzymes and lipoperoxidation status in children with Down syndrome. Clin Biochem.

