# Supplementary material for: Aberrations in circulating inflammatory cytokine levels in patients with Down syndrome: a meta-analysis
Source: Oncotarget. 2017 Sep 19;8(48):84489–96. doi: 10.18632/oncotarget.21060 (PMC5663613; doi:10.18632/oncotarget.21060)
Supplement: Supplementary file 1 [file oncotarget-08-84489-s001.pdf]

Aberrations in circulating inflammatory cytokine levels in patients with Down syndrome: a meta-analysis

SUPPLEMENTARY MATERIALS

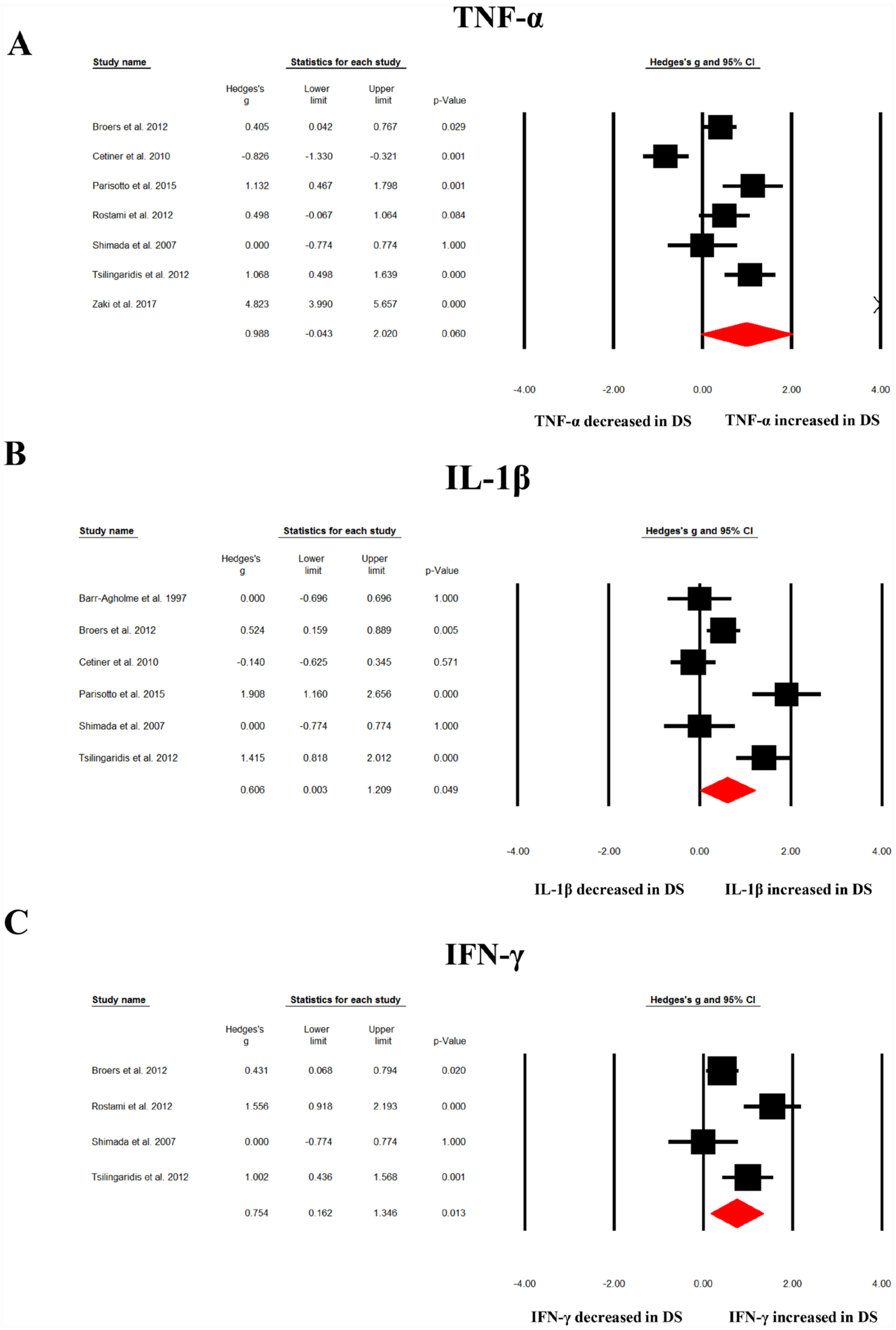

Supplementary Figure 1: Forest plot displaying random effects meta-analysis results of the association between TNF-α (**A**), IL-1β (**B**), IFN-γ (**C**) and children with DS. The sizes of the squares are proportional to study weights.

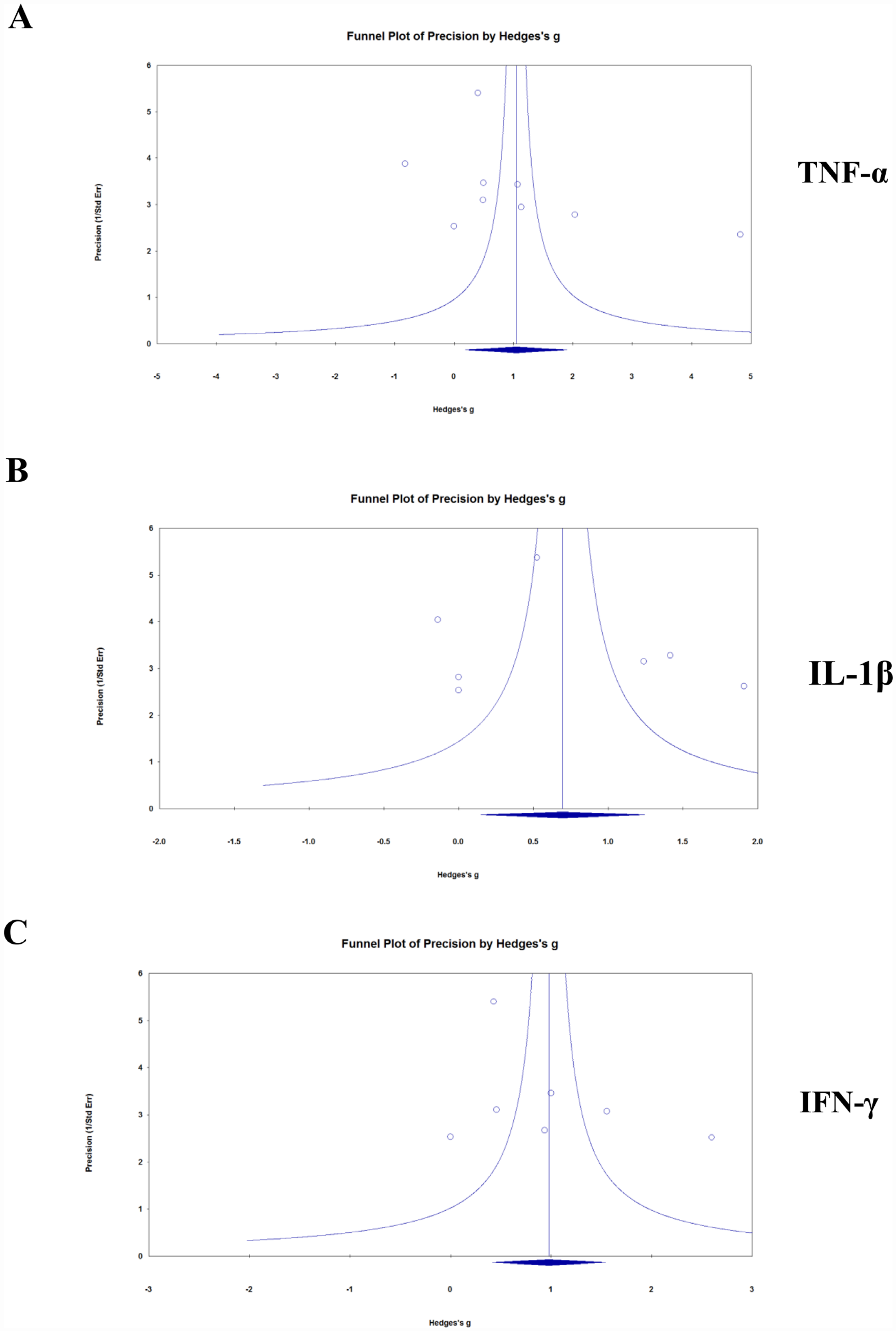

Supplementary Figure 2: Funnel plots examining publication bias in studies comparing TNF-α (**A**), IL-1β (**B**), IFN-γ and (**C**) levels between DS patients and controls.
